# Macrophage migration inhibitory factor urinary excretion revisited – MIF a potent predictor of the immunosuppressive treatment outcomes in patients with proliferative primary glomerulonephritis

**DOI:** 10.1186/s12865-015-0112-1

**Published:** 2015-08-14

**Authors:** Rafał Zwiech

**Affiliations:** Dialysis Department, Barlicki Memorial Teaching Hospital No1, Medical University of Łódź, Kopcińskiego 22, 90-153 Łódź, Poland

## Abstract

**Background:**

Macrophage migration inhibitory factor (MIF) is a cytokine that shares many activities with other pro-inflammatory cytokines in primary glomerulonephritis (GN). This study assesses the influence of immunosuppressive treatment on serum and urine MIF in patients with proliferative (PGN) and non-proliferative (NPGN) glomerulonephritis, and evaluates the potential of MIF in predicting outcomes.

**Methods:**

Eighty-four patients (45 males and 39 females) with primary GN were included. Urinary excretion of MIF (ng/mg of urinary creatinine) was measured both pre- and post-treatment with combined steroids and cyclophosphamide. After a 12-month follow-up, the patients were retrospectively divided into four subgroups: responders of proliferative GN (R-PGN), non-responders of proliferative GN (NR-PGN), responders of non-proliferative GN (R-NPGN) and non-responders of non-proliferative GN (NR-NPGN).

**Results:**

The median pre-treatment urinary MIF values were higher in PGN than in NPGN (3.6 versus 2.2; ANOVA *P* = 0.039). The highest pre-treatment urinary excretion of MIF was observed in NR-PGN (median 6.1), which was significantly higher than other subgroups (ANOVA *P* < 0.05). The treatment significantly reduced MIF urinary excretion only in R-PGN (*P* < 0.01). In NR-PGN, pre- (5.9 ± 2.9 pg/mgCr) and post-treatment mean MIF excretion (4.9 ± 2.3 pg/mgCr) exceeded the calculated cut off value (3.3 pg/mgCr).

**Conclusion:**

MIF urinary excretion appears to be a prognostic marker of therapy outcomes only in proliferative glomerulonephritis, in which lower urinary MIF may be linked with good prognosis, whereas a higher MIF urinary excretion value was a marker of unfavorable therapy outcomes. In Non-Responders, urinary MIF measurements may help to reconsider the choice of the immunosuppressive regimen at early stages of the treatment and act as an impulse to search for new therapeutic strategies.

## Background

Macrophage migration inhibitory factor (MIF) is a cytokine that shares many activities with other pro-inflammatory cytokines, and activates macrophages by promoting the synthesis of such cytokines as tumor necrosis factor-*α*, interleukin (IL)-1*β*, and IL-8 [1–3]. Together with these cytokines, it plays a pivotal role in mediating renal injury in experimental nephritis, including glomerulonephritis. The upregulation of MIF in the glomerular mesangial cells and tubular epithelial cells has been found to correlate with progressive renal injury [4–6].

Furthermore, H.Y. Lan et al. report that MIF expression examined by *in situ* hybridization of specimens obtained from kidney biopsies of various types of primary glomerulonephritis (GN) is markedly upregulated in proliferative forms of human GN [4]. In an experimental GN model, anti-MIF treatment has been found to ameliorate kidney injury, limiting the activation of effector cell, particularly macrophages, and influencing the relationship between infiltrating and local cells [7, 8].

Primary glomerulonephritis, with or without the infiltration of inflammatory cells, is regarded as an immune-mediated disease affecting both the glomeruli and interstitium [9]. Proliferative (PGN) and non-proliferative (NPGN) types of GN were historically differentiated by the presence of leukocyte infiltration, cell proliferation and an imbalance of glomerular extracellular matrix turnover, i.e., the predominance of its production or degradation, as well as their involvement in the injury of kidney structures [10, 11]. Types of proliferative primary glomerulonephritis include mesangial proliferative GN (MesGN), membrano-proliferative GN (MPGN) and immunoglobulin A nephropathy (IgAN), while types of non-proliferative GN include minimal change disease (MCD), focal glomerulosclerosis (FSGS), and membranous nephropathy (MN) [10, 11].

Immunosuppressive therapy (IS) based on the use of corticosteroids and non-specific cytotoxic agents commonly administered in GN remains the elementary therapeutic weapon for immune modification in primary glomerulonephritis. However, this treatment, which modifies immune glomerular injury, remains nonspecific, with a high incidence of side effects and only moderate efficacy [8, 12–17].

Due to the unpredictable course of GN and response to therapy, treatment individualization and optimization is often impossible. Hence, the search continues for more reliable predictors which might allow individually tailored and adequate immunosuppressive treatment schemes to be implemented. This study was therefore conducted to determine whether in severe courses of GN, MIF may be regarded as a complementing factor when deciding to introduce aggressive treatment in sensitive cases, or whether it can be disqualified as a factor preventing IS complications. The objectives of the study were, therefore:

- to compare patients with primary GN and healthy participants with regard to the serum concentration and urinary excretion of MIF,
- to assess the influence of immunosuppressive treatment on the serum and urine MIF levels in patients with proliferative and non-proliferative primary GN and,
- to evaluate the potential of using MIF serum concentration and urine excretion measurements to predict the response to immunosuppressive treatment.

## Methods

### GN patients

The study was conducted in 84 patients (45 male and 39 female) with primary glomerulonephritis: mean age 41.44 ± 13.25 years. The control group consisted of 18 age-matched healthy subjects. The profiles of the study group, including glomerulonephritis-type divisions, and the control group are shown in Table 1. Only patients with a severe course of GN were included in this study, i.e., those who demonstrated deterioration of kidney function, reflected in an increase of serum creatinine concentration by more than 50 % of baseline values or nephrotic range proteinuria, despite steroid treatment. The diagnosis was established based on clinical symptoms, laboratory tests, kidney biopsy and accessory investigations. All of the biopsy specimens were evaluated by light, electron and immunofluorescence microscopy in the Department of Kidney Pathology, Medical University of Lodz. In addition, the percentage of interstitial volume was calculated using a computerized morphometric analysis of interstitium quantity. According to a scoring system proposed by G. Fuiano et al., the degree of glomerulosclerosis (GSC), based on the number of involved glomeruli, and the intraglomerular extent of sclerosis were semi-quantitatively estimated [18–21]. The degree of GSC was graded on a four-point scale: normal (0), mild (1), moderate (2) and severe (3). The type of primary glomerulonephritis was identified by microscopic evaluation, and mesangio-proliferative GN was diagnosed after the exclusion of other types of proliferative glomerulonephritis. Also, the urinary excretion of proteins, expressed in milligrams per milligram of creatinine in urine (mg/mg Cr) was measured, together with total serum protein content.

**Table 1:** The structures of the study group (including subdivisions) according to primary glomerulonephritis type, and the control group

	Gender		Age (years) ± SD	Subgroup	
	Male	Female	Mean	Responders	Non-responders
Non-Proliferative GN					
MCD	3	7	36.0 ± 16.47	8	2
FSGS	9	3	40.83 ± 13.17	7	5
MN	5	6	45.81 ± 13.44	7	4
Proliferative GN					
MesGN	8	12	39.45 ± 15.34	13	7
IgAN	10	10	39.80 ± 14.20	12	8
MPGN	10	1	42.27 ± 11.88	6	5
pooled	45	39	41.44 ± 13.25	53	31
Healthy	10	8	36.11 ± 13.29	-	-

All participants were treated with antihypertensive drugs (angiotensin-converting enzyme inhibitor - ramipril or angiotensin receptor blocker – losartan and calcium channel blocker - amlodipine) to establish and maintain blood pressure values in an accordance with recommendations [22, 23]. In addition, statins were introduced to stabilize the lipid profile (atorvastatin at a mean dose of 20 mg/day) with the aim of reducing low density lipoprotein (LDL) serum concentration to below 100 mg/dl [24]. These variables were assessed at baseline and then serially throughout the course of treatment to monitor the status of the patients. Finally, all tests were repeated after 1 year (±3 months) of immunosuppressive treatment. The structure of the subgroups, divided according to type of primary glomerulonephritis, is presented in Table 1.

### IS protocol

All subjects received an identical immunosuppressive protocol which consisted of initial pulse therapy with methylprednisolone, i.e., a calculated aggregate dose of 1000 mg per 20 kg body weight administered every other day, followed by oral prednisone (25–30 mg/day) and cyclophosphamide in 6 monthly pulses given at 0.6 g/b.m^2^. The cumulative dose of cyclophosphamide did not exceed 6 g. Before the initiation of treatment, the potential foci of infection were diagnosed and eliminated as routine in all participants. A treatment scheme based on pulses of steroids and cyclophosphamide (CPH) was restricted to cases of progressive GN with a severe disease course (decreasing eGFR) and was chosen as a rescue protocol. The introduction of cyclophosphamide as primary treatment is controversial in different types of GN, despite many authors describing it as offering greater benefits than other immunosuppressive agents. CPH pulse treatment combined with steroids is regarded as well tolerated and effective, especially in steroid-dependent or corticosteroid-resistant severe nephrotic syndrome, or in GN with progressive worsening of eGFR and diffuse microscopic lesions, irrespective of primary glomerulonephritis type [25–35].

### MIF analysis

The blood and urine of healthy participants, as well as pre- and post-treatment blood and urine samples from patients, were prospectively collected to EDTA (ethylenediaminetetraacetic acid) tubes, centrifuged and stored at −70 °C until analysis. When completed, the serum concentrations and urinary excretion of MIF were measured by enzyme-linked immunosorbent assay (ELISA) using commercial immunoassays according to the manufacturer’s instructions (Biosource® Europe S.A., Nivelles, Belgium). All tests were performed in the Department of Medical Laboratory Diagnostics, Barlicki Memorial Teaching Hospital No.1, Medical University of Lodz: a certified RIQAS local laboratory (Randox International Quality Assessment Scheme and SNCS IQAS e-CHECK - Sysmex International Quality Assurance System).

To evaluate the potential value of MIF in predicting GN outcomes after 1 year of treatment, patients with PGN and NPGN were divided retrospectively into subgroups according to their response to the therapy: R – Responders (proteinuria < 0.5 g/day, e.g., < 6 mg/mg urine Cr and improved or stable kidney function – serum creatinine change within a range of 15 %), NR – Non-Responders (proteinuria > 0.5 g/day, e.g., > 6 mg/mg urine Cr and/or deterioration of kidney function – over 15 % increase of serum creatinine concentration). These are in accord with previous study methodology [36, 37]. When completed, the division allowed MIF to be evaluated retrospectively at baseline in R and NR.

To verify the value of MIF measurements in predicting GN-favorable outcomes, as well as to confirm the value of abnormal MIF urine excretion in indicating worse pre-treatment prognosis, the cut off value was calculated using an ROC curve.

### Statistical analysis

Comparisons within and between groups were made with the non-parametrical Kruskal-Wallis ANOVA for multivariable analysis, the Fisher’s exact probability test was used for sex comparison and the Wilcoxon’s rank sum test to evaluate changes of clinical parameters during the treatment. Relations between variables were analyzed by Spearman rank (R) correlation coefficients. A logistic regression was performed to analyze potential confounders in the cohort. Differences were considered significant for *p* < 0.05. The results were expressed, as mean ± standard deviation or median (range) as appropriate.

### Ethics statement

The study protocol was approved by the Medical University of Łódź Bioethics Committee, Resolution No. RNN/9/04/KE. According to the principles of good clinical practice (GCP), informed consent was obtained from all patients prior to their inclusion in the study.

## Results

### Clinical data

The clinical data, grouped according to subgroup structure, type of primary glomerulonephritis and patient response to IS, is presented in Table 2. No differences in distribution were noticed between the R and NR subgroups, nor were any differences found with regard to coexisting comorbidities, including diabetes, obesity or severe infections, which might affect the final evaluation in both subgroups. The gender structure in the subgroups was homogenous, and the subgroups were matched according to sex and age.

**Table 2:** Biopsy findings and biochemical parameters in proliferative and non-proliferative glomerulonephritis patients after division into Responders and Non-Responders subgroups (mean ± SD)

	Proliferative GN		Non-proliferative GN	
	Responders (R)	Non-Responders (NR)	Responders (R)	Non-Responders (NR)
Patients (n)	31	20	22	11
Uncontrolled hypertension (n)	3	2	2	2
ACE-I or ARB treatment(n)	29	18	21	11
Interstitium volume (%) ± SD	30.3 ± 2.27^b,c^	31.1 ± 3.11^d,e^	14.2 ± 5.47^b,d^	15.03 ± 7.35^c,e^
Median	22.3	21.5	16.7	18.3
Glomerulosclerosis (grade) ± SD	1.65 ± 0.81	1.68 ± 0.74	2.66 ± 0.51	2.59 ± 0.61
Median	1.88	1.99	2.24	2.15
Mesangial cellularity	1.45 ± 0.28	1.39 ± 0.19	0.96 ± 0.11	0.87 ± 0.19
Median	1.22	1.19	0.99	0.91
Serum creatinine before treatment (mg %) ± SD	1.88 ± 1.01	1.86 ± 1.1	1.45 ± 1.19	1.66 ± 1.4
Median	1.75	1.73	1.59	1.61
Serum creatinine after treatment (mg %) ± SD	1.40 ± 1.42^a,c^	2.17 ± 1.61	1.23 ± 1.14^d,f^	2.39 ± 1.74
Median	1.39	1.93	1.24	2.27
LDL before treatment (mg/dl) ± SD	126 ± 41.1^a,b,c^	139 ± 39.7	145.7 ± 39.1	146.8 ± 41.7
Median	122.2	137.5	141.7	142.6
LDL after treatment (mg/dl) ± SD	87.9 ± 10.4	88.8 ± 11.1	88.1 ± 9.9	89.5 ± 11.6
Median	86.6	87.9	88.0	88.4
Total serum proteins before treatment (g/dl) ± SD	6.02 ± 0.55	6.11 ± 0.58	5.51 ± 1.82	5.48 ± 1.78
Median	5.88	5.91	5.55	5.69
Total serum proteins after treatment (g/dl) ± SD	6.42 ± 0.62^a,c^	5.64 ± 0.81^a,d^	6.65 ± 0.6^d,f^	5.24 ± 0.89^c,f^
Median	6.56	5.54	6.74	5.31
Proteins urine excretion before treatment (mg/mg Cr) ± SD	11.4 ± 2.44^b,c^	12.1 ± 6.08^d,e^	61.25 ± 9.4^b,d^	58.90 ± 88.18^c,e^
Median	10.5	11.0	58.5	51.3
Proteins urine excretion after treatment (mg/mg Cr) ± SD	1.58 ± 3.1^a,b,c^	2.96 ± 9.14^a,e^	5.55 ± 2.25^b,f^	19.9 ± 14.3^b,c,e,f^
Median	1.31	2.66	3.88	16.74

Differences were considered significant for *P* < 0.05 (Kruskal-Wallis ANOVA)

^a^Proliferative GN R versus NR

^b^Proliferative GN R versus non-proliferative GN R

^c^Proliferative GN R versus non-proliferative GN NR

^d^Proliferative GN NR versus non-proliferative GN R

^e^Proliferative GN NR versus non-proliferative GN NR

^f^Non-proliferative GN R versus NR

As shown in Table 2, no statistically significant differences in biopsy findings, such as percentage of interstitium volume and glomerulosclerosis grade, or biochemical parameters, such as total serum protein content and serum creatinine concentration, were observed between R and NR subgroups in either type of GN for all comparisons before treatment. In proliferative GN, the only parameter which differed between both subgroups was the presence of a significantly reduced pre-treatment LDL serum concentration in the R subgroup. In both subgroups of non-proliferative GN, pre-treatment urine excretion of protein was significantly greater than in proliferative GN. Before treatment, significant positive correlations were found between serum creatinine concentration and interstitium volume (*ρ* = 0.263, *P* = 0.029), and glomerulosclerosis grade (*ρ* = 0.294, *P* = 0.036) in proliferative glomerulonephritis. Also, in non-proliferative primary glomerulonephritis, a positive correlation was also noted between serum creatinine and interstitium volume (*ρ* = 0.28, *P* = 0.043), and glomerulosclerosis grade (*ρ* = 0.22, *P* = 0.038).

When analyzing the R and NR subgroups for both proliferative and non-proliferative primary glomerulonephritis during the post-treatment period, total serum protein level was significantly higher in the R subgroup, while, as expected, serum creatinine concentration and protein urine excretion were significantly lower in the R subgroup, and followed from assumed subdivision to Responders and Non-Responders However, no difference in post-treatment protein urine excretion was observed between the NR proliferative and R non-proliferative glomerulonephritis. The introduction of statins resulted in post-treatment LDL reduction, and serum LDL concentrations did not differ significantly between the proliferative and non-proliferative GN groups for both the R and NR subgroups. The results are presented in Table 2.

### Serum MIF

Serum MIF levels before treatment did not differ significantly between proliferative and non-proliferative primary GN patients. The MIF serum concentration in patients with both types of GN were significantly higher than in healthy subjects (*P =* 0.021 and *P* = 0.036, respectively). The results are presented in Table 3. No difference was seen between the R and NR subgroups with regard to pre-treatment MIF serum concentration, irrespective of whether proliferative or non-proliferative primary glomerulonephritis was analyzed (Table 4). Although, the post-treatment mean serum MIF values were slightly lower in proliferative GN, both for the R versus NR and in non-proliferative GN for the R versus NR subgroups (i.e., 1.1 ± 0.7 vs. 2.4 ± 1.1 and 1.6 ± 0.8 vs.1.8 ± 0.9 ng/ml, respectively), the observed reductions were not statistically significant. With respect to pre and post-treatment MIF, no correlations between serum concentration and other cofounders were found, irrespective of the type of primary glomerulonephritis.

**Table 3:** Pre-treatment serum concentration and urinary excretion of MIF in patients with proliferative and non-proliferative glomerulonephritis (GN)

		Serum MIF (ng/ml)	Urinary MIF (ng/mg Cr)
Proliferative GN	Mean ± SD	2.2 ± 1.0	4.2 ± 2.1
	Median (range)	1.9 (1.1–3.4)	3.6^a,b^ (2.0–8.1)
Non-proliferative GN	Mean ± SD	1.8 ± 0.3	3.0 ± 1.9
	Median (range)	1.3 (1.0–2.4)	2.2^c^ (1.7–4.3)
Pooled	Mean ± SD	1.7 ± 0.8	3.7 ± 1.9
	Median (range)	1.7 (1.0–3.3)	3.2 (1.7–8.1)
Healthy	Mean ± SD	0.2 ± 0.01	0.5 ± 0.02
	Median (range)	0.2 (0.18–0.21)	0.5 (0.4–0.5)

Differences were considered significant for *P* < 0.05 (Kruskal-Wallis ANOVA)

^a^Proliferative GN versus Non-proliferative GN

^b^Proliferative GN versus Healthy patients

^c^Non- proliferative GN before versus Healthy

**Table 4:** Pre-treatment serum concentration and urinary excretion of MIF in patients with proliferative and non-proliferative glomerulonephritis (GN) after division into Responders (R) and Non-Responders (NR) subgroups

		Serum MIF (ng/ml)		Urinary MIF (ng/mg Cr)	
		Mean ± SD	Median (range)	Mean ± SD	Median (range)
Proliferative GN	R	1.3 ± 0.8	1.4 (1.1–1.5)	2.1 ± 1.3	2.4 (2.0–3.7)
	NR	3.3 ± 1.2	3.0 (2.0–3.4)	5.9 ± 2.9	6.1^a,b,c^ (2.9–8.1)
Non-proliferative GN	R	1.7 ± 0.2	1.8 (1.0–2.0)	2.9 ± 1.8	2.2 (1.7–4.1)
	NR	2.0 ± 0.4	1.9 (1.2–2.4)	3.1 ± 2.1	2.3 (1.9–4.3)

Differences were considered significant for *P* < 0.05 (Kruskal-Wallis ANOVA)

^a^Proliferative GN NR versus proliferative GN R

^b^Proliferative GN NR versus non-proliferative GN R

^c^Proliferative GN NR versus non-proliferative GN NR

### Urinary MIF

Greatly increased MIF urinary excretion was observed in patients with proliferative and non-proliferative primary glomerulonephritis compared to controls (*P* = 0.006 and *P* = 0.009 – Table 3). The urinary excretion of MIF before treatment positively correlated with interstitium volume, glomerulosclerosis grade and serum creatinine concentration: *ρ* = 0.29, *P* = 0.036; *ρ* = 0.24, *P* = 0.038; *ρ* = 0.31, *P* = 0.039, respectively.

**Fig. 1:**
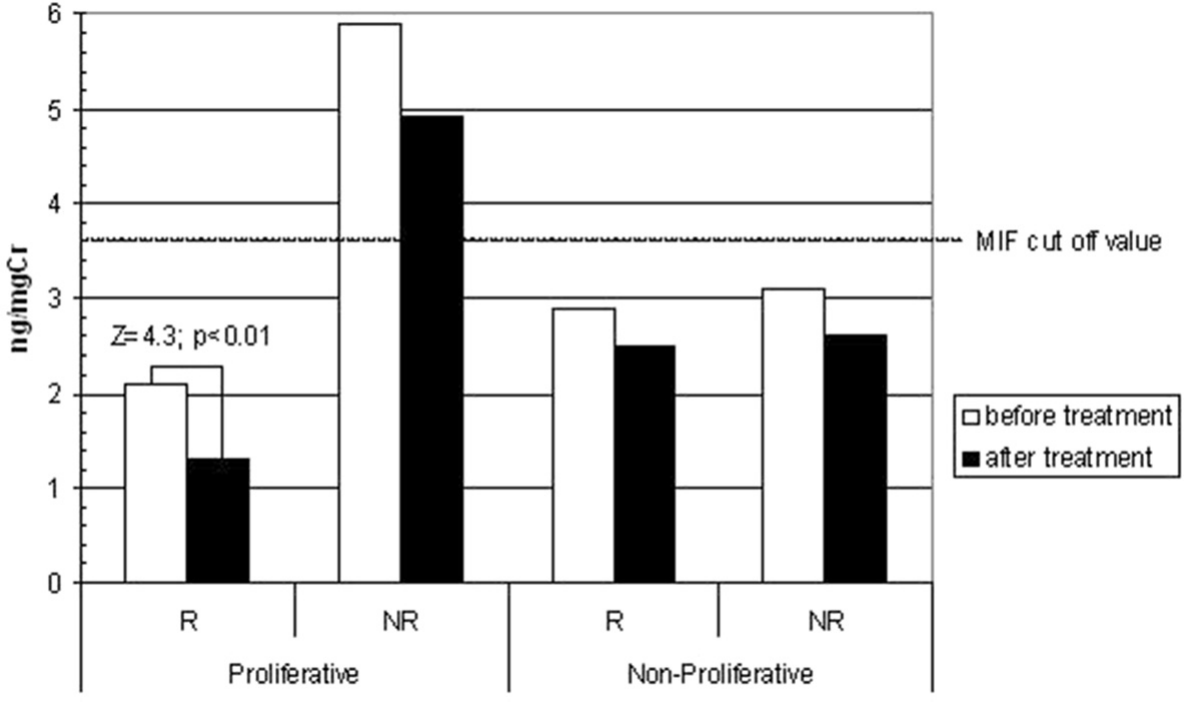
Pre-treatment and post-treatment mean urinary excretion of MIF in proliferative and non-proliferative glomerulonephritis (GN) divided into Responder (R) and Non-Responder (NR) subgroups

Urinary MIF was higher in PGN than in NPGN (*P* = 0.039). The treatment reduced urinary MIF in both primary glomerulonephritis types, irrespective of the response to treatment (R and NR subgroups), but MIF urinary excretion was only found to be significantly lower in Responders with PGN (*P* = 0.007) – Fig. 1. All results are presented in Table 3. The pre-treatment MIF urinary excretion value in PGN positively correlated with interstitium volume, glomerulosclerosis grade and serum creatinine concentration: *ρ* = 0.28, *P* = 0.03; *ρ* = 0.3, *P* = 0.037; *ρ* = 0.34, *P* = 0.026; respectively.

In non-proliferative GN, MIF urinary excretion did not differ between the R and NR subgroups. In contrast, in proliferative glomerulonephritis, the urinary excretion of MIF was significantly higher in the NR than the R subgroup (Table 4). It is noteworthy that MIF excretion in the R subgroup was significantly greater than that observed in the R and NR subgroups in non-proliferative GN. Interestingly, urinary MIF levels in the Responders subgroup of proliferative GN were found to be similar to those of both the R or NR subgroups in non-proliferative GN (Table 4).

The calculated ROC curve indicated the cut-off value for urinary MIF excretion as 3.3 pg/mg of urinary creatinine, and the AUC was 0.87 (95 % CI 0.8–0.91). Only the urinary excretion of MIF in Non-Responders in proliferative primary GN was higher than the calculated pre- (*P* = 0.009) and post-treatment (*P* = 0.013) cut-off values. Furthermore, in no other subgroup was this limit exceeded, neither before nor after IS treatment (Fig. 1). The evaluation of pre-treatment urinary MIF values in Non-Responders (proliferative GN) revealed a correlation with post-treatment serum creatinine (*ρ* = 0.42, *P* = 0.006) and proteinuria values (*ρ* = 0.44, *P* = 0.005). The logistic regression analysis of the pre-treatment variables indicated that only the urinary MIF was the exclusive predictive marker of the treatment outcomes in the whole cohort Table 5.

**Table 5:** Multiple logistic regression analysis of the pre-treatment variables which may influence the response to the immunosuppressive treatment (the whole cohort of Responders)

	Estimation	Odds ratio	95 % CL	*P*-value
Interstitium volume	−0.02	0.97	0.91–1.02	NS
Glomerulosclerosis (grade)	−0.23	0.78	0.06–9.6	NS
Serum creatinine	0.63	1.8	0.85–4.1	NS
LDL	0.3	0.9	0.7–1.31	NS
Proteins urine excretion	−0.03	0.96	0.92–1.0	NS
ACE-I, ARB treatment	0.12	1.12	0.22–5.62	NS
Serum MIF	0.04	1.3	0.99–1.6	NS
Urinary MIF	−0.41	0.21	0.11–0.64	0.022

## Discussion

Macrophage migration inhibitory factor (MIF) is a potent pro-inflammatory cytokine which has been found to have an important chemokine-like function (CLF) which plays an essential role in monocyte recruitment and arrest; It has also been recently redefined as a pleiotropic inflammatory cytokine which has crucial roles in both physiological immunity and inflammatory diseases [1, 38–41]. Renal MIF expression was identified in normal kidneys and it is upregulated in patients with glomerulonephritis and renal allograft rejection. This upregulation of MIF in GN is typically associated with leukocyte infiltration, histopathological damage and kidney dysfunction in patients with inflammatory kidney disease [1, 4, 40, 41]. Most authors assume increased urinary MIF excretion to be significantly correlated with local – renal MIF upregulation irrespective of its serum level, which suggests that MIF may be a suitable candidate as a marker for renal injury in humans [41, 42]. K. Matsumoto et al. report MIF urinary excretion to be significantly correlated with the grades of mesangial matrix increase and interstitial fibrosis, as well as with the number of both intraglomerular and interstitial macrophages among patients with focal glomerular sclerosis [43]. The authors also exclude any possible correlation between the levels of urinary MIF and the severity of proteinuria, which suggests that the detectability of MIF in urine reflects a specific involvement in the disease process of GN [43]. Furthermore, anti-MIF intervention in experimental glomerulonephritis has revealed a generalized downregulation of numerous pro-inflammatory cytokines, chemokines and MIF-dependent signaling intermediates [7, 8]. These conclusions are in agreement with observations concerning the protective effect of genetic MIF deficiency on renal injury reported in MRL/MpJ-Faslpr mice backcrossed onto a mif−/− background [40].

In the present study, only participants with severe proteinuria and/or an increase of serum creatinine were selected. Unsuccessful initial treatment with steroid therapy (i.v. pulses) necessitated the need for combined treatment with cyclophosphamide. Although this immunosuppressive protocol exceeds the scope of standard therapy, many authors confirm its efficacy in various types of primary glomerulonephritis [25–35]. However, the patients to be treated by this therapeutic strategy must be carefully selected i.e., those who are known to have a severe course of GN. In this study, one third of non-proliferative glomerulonephritis and nearly 40 % of patients with proliferative GN demonstrated no improvement in proteinuria and/or kidney function after therapy and continued to 1 year follow-up, despite combined treatment. This appears to confirm that the clinical course of GN still remains unpredictable in a significant number of patients, and that it is hard to form a uniform therapeutic strategy. Furthermore, in some cases, treatment options based on routine clinical and biochemical assessments can be introduced, but often in vain [44, 45]. As the initial introduction of less radical immunosuppressive schemes may be insufficient, more aggressive therapeutic strategies in those particularly complicated cases merit further investigation [12–16].

In the present study, no differences were noted between pre- and post-treatment MIF serum concentrations, irrespective of the type of glomerulonephritis. Highly elevated serum MIF values seem to be associated with a significant deterioration of kidney function in the majority of participants, although the values were even lower than those of MIF urinary excretion.

Urinary MIF excretion has been occasionally evaluated [3, 43]. In this study, urinary MIF was found to be lower after treatment in both the proliferative and non-proliferative GN groups. R. Bucala reports that the low expression of MIF alleles may protect the end-organ from ensuing inflammatory damage, and the immunosuppressive action of glucocorticoids may be most effectively applied in those individuals who, on the basis of their genotype, manifest an MIF-dependent form of autoimmunity [17].

However, significant reductions were demonstrated only in PGN participants who responded to treatment. On the contrary, K. Matsumoto et al. report that MIF urinary excretion in patients with focal glomerular sclerosis was significantly depressed immediately following administration of isolated steroid therapy [43]. This trend was also observed for Responders with PGN in the present study, but only after the combined therapy was administered. Hence, although the decrease in MIF urinary excretion after the introduction of immunosuppressive therapy seems to be a good prognosis of favorable treatment outcome, it is not the case in all patients with GN, but only in participants with proliferative glomerulonephritis and in whom this reduction was statistically significant. Therefore, the significant reduction in urinary MIF excretion may reduce the synthesis of a pathway of pro-inflammatory cytokines and decreases macrophages activity in both the glomeruli and interstitium [7, 8].

It is noteworthy that MIF was the only parameter that was found to vary between the Responders and Non-Responders subgroups in the proliferative GN group. In Non-Responders, the MIF urinary excretion values were even higher than those seen in patients described by K. Matsumoto et al., or after stimulation with pIgA prepared from IgAN patients [3, 43]. Although all post-treatment urinary MIF values were lower than those seen in pre-treatment in both the R and NR subgroups, the lowest value was still noted in responders with proliferative GN. The present study suggests that lower MIF urinary excretion may be associated with a good prognosis, and increased excretion with the risk of GN progression, which is in accord with other studies, although it was also observed for non-proliferative GN [3–6, 43]. However, MIF plays a crucial role in GN, particularly proliferative types of GN, may be due to its pro-inflammatory activity. Also, in proliferative GN, the correlations between pre-treatment urinary MIF and post-treatment creatinine and proteinuria shown in the present study highlight the potential value of MIF measurements as a marker of poor prognosis. Among the patients with GN, urinary MIF significantly correlates with the grade of mesangial matrix and interstitial fibrosis as well as with both the intraglomerular and interstitial macrophage infiltration rate [43]. However, these may explain the correlations between urinary MIF and the magnitude of proteinuria [44] and degree of renal dysfunction [41]: All those assessments were obtained before the treatment was instituted. A review of extant literature indicates, that the correlations between MIF and post-treatment parameters have not been investigated at the time of writing.

To verify the potency of MIF measurements in predicting GN, the cut off value of MIF urinary excretion was calculated using an ROC curve. The results indicated that MIF urinary excretion was higher only in Non-Responders of proliferative primary GN, both pre- and post-treatment, which is important in the light of the present study. However, although proteinuria is regarded as an independent risk factor and a predictor of renal function deterioration in some types of primary glomerulonephritis, it is not accurate at baseline in some cases [39, 45]. In these cases, creatinine clearance, hypertension and severity of biopsy pathological lesions are key indicators [38, 46]. Surprisingly, in this study, those risk factors traditionally regarded as contributory for treatment outcome were found to be unreliable.

The decision to introduce immunosuppressive therapy is strictly based on patient general status, clinical and biochemical parameters. Additionally, the clinicians individualize the treatment to achieve high effectiveness and avoid therapeutic disadvantages based on their experience and existing guidelines The present findings suggest that pre-treatment examinations of urinary MIF may become an additional factor in the optimization of treatment. Additionally MIF seems to be not only a potent predictive marker but also may add impetus to the search for new therapeutic strategies like anti-MIF treatment which has been found to ameliorate kidney injury in an experimental GN models [7, 8].

Nevertheless, further studies evaluating the value of MIF urinary excretion measurements in GN, especially the proliferative type, are needed.

### Study limitations

As only a selected group of patients with a severe course of GN were evaluated, the number of participants evaluated in this study is relatively small. However, this number is still higher than in most cited studies. Additionally, all participants underwent an immunosuppressive treatment protocol selected by the center and not widely recruited, but this was undoubtedly efficient in severe cases of glomerulonephritis [25–33]. Although these disadvantages may detract from the results of post-treatment MIF evaluation, and a retrospective analysis (R and NR subdivisions) may be presumed to be less significant than a prospective trial, the value of multivariable logistic evaluation using MIF as an exclusive contributory predictor of therapy outcomes should not be underestimated.

## Conclusion

In conclusion, although urinary MIF excretion is highly elevated in patients with primary GN, this serum concentration is not significantly higher than in healthy participants.

The study did not confirm that MIF has any prognostic value in the course of non-proliferative primary glomerulonephritis. In contrast, in participants with proliferative GN, a higher MIF urinary excretion value was a marker of unfavorable therapy outcomes, whereas lower urinary MIF in these patients may be linked with a good prognosis. Additionally, in patients with proliferative GN who positively responded to therapy, the immunosuppressive treatment significantly reduced urinary MIF. In Non-Responders urinary MIF measurements may help to reconsider the choice of the immunosuppressive regimen during the early stage of the treatment and may add impetus to the search for new therapeutic strategies.

## Abbreviations

ACE-I: Converting enzyme inhibitor
ARB: Angiotensin II receptor blocker
CPH: Cyclophosphamide
ELISA: Enzyme-linked immunosorbent assay
FSGS: Focal segmental glomerulosclerosis
GCP: Good clinical practice
GN: Glomerulonephritis
GN: Glomerulonephritis
GSC: Glomerulosclerosis
IgAN: Immunoglobulin A nephropathy
IS: Immunosuppressive therapy
LDL: Low density lipoprotein
MCD: Minimal change disease
MesGN: Mesangio-proliferative glomerulonephritis
MIF: Macrophage migration inhibitory factor
MN: Membranous nephropathy
MPGN: Membrano-proliferative glomerulonephritis
NPGN: Non-proliferative glomerulonephritis
NR: Non-Responders
PGN: Proliferative glomerulonephritis
R: Responders
